# Interferon therapy for preventing COPD exacerbations

**DOI:** 10.17179/excli2020-2997

**Published:** 2020-11-04

**Authors:** Meenu Mehta, Keshav R. Paudel, Shakti D. Shukla, Madhur D. Shastri, Sachin K. Singh, Monica Gulati, Harish Dureja, Gaurav Gupta, Saurabh Satija, Philip M. Hansbro, Dinesh K. Chellappan, Kamal Dua

**Affiliations:** 1 Discipline of Pharmacy, Graduate School of Health, University of Technology Sydney, NSW 2007, Australia; 2 Centre for Inflammation, Centenary Institute, Sydney, NSW 2050, Australia; 3 School of Life Sciences, Faculty of Science, University of Technology Sydney, NSW 2007, Australia; 4 Priority Research Centre for Healthy Lungs, Hunter Medical Research Institute (HMRI), University of Newcastle, New Lambton Heights, Newcastle, NSW 2305, Australia; 5 School of Health Sciences, College of Health and Medicine, University of Tasmania, Launceston, Australia; 6 School of Pharmaceutical Sciences, Lovely Professional University, Phagwara, Punjab, India; 7 Department of Pharmaceutical Sciences, Maharshi Dayanand University, Rohtak, Haryana, India; 8 School of Pharmacy, Suresh Gyan Vihar University, Jagatpura, Jaipur, India; 9 Department of Life Sciences, School of Pharmacy, International Medical University (IMU), Kuala Lumpur, Malaysia; 10 School of Pharmaceutical Sciences, Shoolini University, Solan, Himachal Pradesh, India

## ⁯⁯⁯

***Dear Editor, ***

Interferons (IFNs) are a group of secreted autocrine and paracrine proteins released by host cells that impart resistance against viral infections. IFNs that are produced by virus-infected cells help other cells in the proximity to heighten their anti-viral defences. Based on their affinity to bind with various receptors, IFNs are classified into three classes, namely, Type I, Type II, and Type III IFNs (Borden et al., 2007[2]). These unique signaling proteins facilitate the transcription of more than 300 IFN-inducible genes, which are also referred to as IFN-stimulated genes (ISGs), that actively participate in the process of viral protein synthesis inhibition (Hsu et al., 2015[8]).

Chronic obstructive pulmonary disease (COPD) is the third leading cause of death worldwide. In a given population aged more than 45 years, COPD affects at least 10 % of them, whereas, it affects more than 50 %, among chronic smokers (Chellappan et al., 2020[3], Mehta et al., 2020[13]). Chronic airway inflammation in COPD is mediated by pro-inflammatory cytokines which include several IFNs (Chin et al., 2020[4]). Furthermore, the expression levels of interferon stimulated genes - myxovirus resistance gene A (ISG MxA), 2'-5'-oligoadenylate synthase 1, and viperin genes are reduced in severe COPD patients. 

Influenza A virus (IAV) infected human primary airway epithelial cells (hPECs) isolated from active smokers have shown impaired antiviral responses compared to hPECs isolated from non-smokers. IAV triggers the release of an array of IFNs, primarily, IFN-λ 1, IFN-λ 2/3 and IFN-β, which in turn stimulate a robust antiviral response in hPECs from non-smokers. In contrast, hPECs from smokers showed inhibition of IFN-λ1, IFN-λ 2/3 and IFN-β mRNA responses by 96 %, 95 % and 85 %, respectively (Wu et al., 2016[15]). Moreover, there is a downregulation of IFN-β signaling in the cell-free sputum supernatant of smokers, when compared to people who have never smoked. 

Cigarette smoke extract (CSE) may activate serine phosphorylation driven ubiquitination and degradation of the IFN alpha/beta receptor 1 subunit of the Type I IFN receptor. This may lead to reduced IFN signaling cascade and a corresponding amount of suppression in the resistance against viral infections (HuangFu et al., 2008[9]). Therefore, COPD patients are likely to experience acute infectious exacerbations leading to an increased risk of mortality/morbidity, as well as, reduced quality of life and poor prognosis. Moreover, immunolabeling scores of IFN-β and its main transcription factor (IRF-7) were reduced up to 65 % and 74 % respectively, in epithelial tissues and alveolar macrophages in COPD patients, when compared with non-COPD patients (Garcia-Valero et al., 2019[7]). 

The use of experimental approaches involving *in vitro* cell culture systems or *in vivo* animal models have revealed key mechanistic insights, which support the fact that, cigarette smoke dampens Type I IFN responses followed by an exaggerated inflammatory response to viral infections. An *in vivo* investigation that employed the elastase-induced experimental emphysema mouse model with C57BL/6J and BALB/cJ mouse strains has reported that, the BALB/cJ mice had high levels of interleukin (IL)-17A mRNA and a number of classically (M1) and alternatively (M2) activated macrophage genes, whereas, C57BL/6J mice showed high levels of IFN-γ. This study demonstrated that mice with different genetic backgrounds show contrasting susceptibility to the progression of emphysema, which is a common feature in COPD (Limjunyawong et al., 2015[10]).

Increased airway inflammation was observed in mice exposed to cigarette smoke followed by chronic infection with non-typeable *Haemophilus influenzae *(NTHi) virus compared to air-exposed controls. NTHi-specific T cells from cigarette smoke-exposed mice generated lower levels of IFN-γ and IL-4 (Lugade et al., 2014[11]). IFN-γ may modulate macrophages to increase the response to toll-like receptor (TLR) ligands, such as LPS. IFN-γ increases the expression levels of TLR2 and TLR4. It further activates the Janus Kinase (JAK)/Signal Transducer and Activator of Transcription (STAT) signaling pathway in alveolar macrophage. IFN-γ mediated cytokine-expression and STAT1 activation were resistant to corticosteroids (dexamethasone) while, JAK/STAT1 inhibition repressed the effect of IFN-γ. Therefore, targeting IFN-γ signaling by JAK inhibitors could be a promising novel therapeutic approach against COPD.

In an *ex vivo* study conducted with bronchoalveolar lavage (BAL) cells (95 % were macrophages) collected from 7 COPD patients and 10 healthy control subjects, the cells infected with live rhinovirus resulted in a 50 % impairment in IFN-α and IFN-λ release from the COPD group compared to control. Similarly, the level of IFN-β stimulated by rhinovirus was remarkably higher in the healthy control subjects compared with the COPD group. The low levels of IFNs in COPD were correlated with impairment in the induction of the ISG CXCL10 (Mallia et al., 2011[12]). 

Another study that employed primary bronchial epithelial cells isolated from COPD and healthy volunteers, who were infected with Rhinovirus-1B, showed that the cells from COPD patients had a higher expression of interleukin (IL)-6, IFNs-β and λ1 when compared to the cells from healthy subjects. The pre-treatment of IFN-β/λ1 to cells from COPD patients did not change IFN-β expression but resulted in increased levels of IFN-λ1 leading to suppression of viral replication. However, this did not raise pro-inflammatory cytokines (Baines et al., 2013[1]). Moreover,* in vitro *experiments conducted in both human alveolar basal epithelial adenocarcinoma cell line (A549) and primary bronchial epithelial cells showed that, IFN-β exerts not only a robust, but also a prolonged protective effect against Rhinovirus infection. The viral RNA was found to be reduced in both cell lines after 18 hours of exposure with IFN-β followed by infection with rhinovirus for the next 72 hours (Gaajeetaan et al., 2013[6]). 

Similarly, in a lung organ culture model, CSE exposure led to inhibition of IFN-inducible protein-10, and mRNA expression. Similarly, there was a reduction of IFN-β mRNA by CSE. This suppression was due to oxidative inhibition of viral-mediated induction of the pattern recognition receptor RIG-I (Wu et al., 2011[14]). Stimulation of the RIG-I/mitochondrial antiviral signaling protein pathway by intracellular delivery of polyinosinic: polycytidylic acid (with liposomes or *via *nucleofection) upregulated IFN-β, mainly through the TLR3/TRIF pathway in primary airway epithelial cells with low co-stimulation of IL-8. This study highlights the feasibility of augmenting the production of IFN-β by airway epithelial cells without excessive co-stimulation of IL-8 (Dauletbaev et al., 2015[5]). 

A collective and comprehensive understanding of the role of IFNs in the pathogenesis of COPD may provide us with a promising platform to understand the in-depth disease mechanism and would provide a logical understanding to investigate potential therapeutic alternatives. 

## Notes

Meenu Mehta and Keshav R. Paudel contributed equally as first author.

## Conflict of interest

None.
